# Nanocarriers Based on Gold Nanoparticles for Epigallocatechin Gallate Delivery in Cancer Cells

**DOI:** 10.3390/pharmaceutics14030491

**Published:** 2022-02-24

**Authors:** Lídia Cunha, Sílvia Castro Coelho, Maria do Carmo Pereira, Manuel A. N. Coelho

**Affiliations:** 1 LEPABE—Laboratory for Process Engineering, Environment, Biotechnology and Energy, Faculty of Engineering, University of Porto, Rua Dr. Roberto Frias, 4200-465 Porto, Portugal; up201406702@edu.fe.up.pt (L.C.); mcsp@fe.up.pt (M.d.C.P.); mcoelho@fe.up.pt (M.A.N.C.); 2 ALiCE—Associate Laboratory in Chemical Engineering, Faculty of Engineering, University of Porto, Rua Dr. Roberto Frias, 4200-465 Porto, Portugal

**Keywords:** drug delivery systems, gold nanoparticles, epigallocatechin gallate, antioxidant activity, pancreatic cancer cells

## Abstract

Gold nanoparticles (AuNPs) are inorganic and biocompatible nanovehicles capable of conjugating biomolecules to enhance their efficacy in cancer treatment. The high and reactive surface area provides good advantages for conjugating active compounds. Two approaches were developed in this work to improve the Epigallocatechin-3-gallate (EGCG) antioxidant efficacy. AuNPs were synthesized by reducing gold salt with chitosan. One other nanosystem was developed by functionalizing AuNPs with cysteamine using the Turkevitch method. The physico-chemical characterization of EGCG conjugated in the two nanosystems-based gold nanoparticles was achieved. The in vitro toxic effect induced by the nanoconjugates was evaluated in pancreatic cancer cells, showing that encapsulated EGCG keeps its antioxidant activity and decreasing the BxPC3 cell growth. A significant cell growth inhibition was observed in 50% with EGCG concentrations in the range of 2.2 and 3.7 μM in EGCG-ChAuNPs and EGCG-Cyst-AuNPs nanoconjugates, respectively. The EGCG alone had to be present at 23 μM to induce the same cytotoxicity response. Caspase-3 activity assay demonstrated that the conjugation of EGCG induces an enhancement of BxPC3 apoptosis compared with EGCG alone. In conclusion, AuNPs complexes can be used as delivery carriers to increase EGCG antioxidant activity in cancer tissues.

## 1. Introduction

Gold nanoparticles are vectors that present the capacity to target biocompounds without loss of their activity and overcome the drawbacks associated with the conventional agents used in cancer treatment. The low bioavailability, stability and half-life time are the main limitations of biocompounds [1,2]. The concept of the presented study is based on the delivery of an antioxidant agent using gold nanoparticles (AuNPs). AuNPs are inorganic vehicles that present unique physical, chemical and optical characteristics [3,4]. Their reactive surface, easy conjugation and biocompatibility provide high loading capacity for drug entrapment and improve its stability in several biomedical applications [3].

Epigallocatechin-3-gallate (EGCG) is the chosen antioxidant in this study. It is a natural green tea compound that possesses powerful antioxidant and anticancer activities [5,6,7,8]. This molecule presents a short half-life, low stability and low bioavailability [6,9]. Several studies reported a significant EGCG effect verified on cancer cells lines and animal model by angiogenesis, apoptosis, and transcription factor [5]. In recent years, AuNPs have been used as a system to protect and deliver drugs/active molecules in situ [5]. The excellent capacity of nanoparticles to modify their surface with targeting ligands allows improving their accumulation in tumors compared to free molecules [6,10]. The delivery of molecules to tumor occurs by passive targeting due to the enhanced permeability and retention (EPR) effect [3]. Safwat et al. demonstrated the good potential of AuNPs to increase anticancer effect of EGCG in Ehrlich ascites carcinoma-bearing mice [6]. Sanna et al. suggested that 25 nm stable EGCGAuNPs present more antioxidant activity when compared with EGCG alone [11]. Additionally, the in vitro cell growth study of EGCGAuNPs assessed against human neuroblastoma SH-SY5Y-derived cells found high toxicity of the nanosystem compared with EGCG alone. Mostafa et al. reported the 35 nm EGCGAuNPs with high toxicity on hepatocellular carcinoma HepG2 cells compared with EGCG alone [12]. The let-7a and miR-34a expression in the cells were analyzed, demonstrating their high expression levels after 72 h of incubation with EGCGAuNPs.

The aim of this study is to develop a drug-delivery nanosystems-based AuNPs with optimal size range diameters (between 100–200 nm) promoting a high cell uptake rate [13,14]. The nanoparticles might be internalized via clathrin- or caveolin-mediated endocytosis [14]. The developed nanoformulations presented opposite charges to incorporate EGCG. The strategy was to synthesize both AuNPs using chitosan as the reducing agent and cysteamine as capping agent [15,16]. Chitosan was chosen to improve the nanosystem biocompatibility [17,18]. Cysteamine was selected to functionalize gold nanoparticles in order to increase the therapeutic effect of the nanosystem [19,20]. The enhancement of the nanosystems efficacy was evaluated. The behavior application of the chitosan–gold nanoparticles (ChAuNPs) and cysteamine–Au NPs (CystAuNPs) as the delivery vehicles of EGCG were studied using the pancreatic adenocarcinoma BxPC3 cell line. EGCG-based AuNPs might increase the solubility and bioavailability of the active compound, prolonging its circulation time and induce to higher levels of inhibition [21].

ChAuNPs were produced by a green synthesis method with chitosan (Ch) as reducing agent. Chitosan is a natural cationic polysaccharide derived from partial chitin deacetylation. It is constituted by β-(1-4)-linked D-glucosamine and N-acetyl-D-glucosamine randomly distributed within the polymer [17,22,23]. Ch is a biocompatible and non-toxic polysaccharide. It presents low allergenicity and biodegradability properties [3,24]. Bhattarai et al. reported the production of N-acylated chitosan-stabilized AuNPs for applications in physiological conditions, exhibiting the advantages of non-acylated chitosan [25]. Several studies demonstrated the antitumor activity of chitosan in in vitro and in vivo models [22,26]. Lin et al. suggested the significant anti-tumorigenesis effect of chitosan against gastric and colon cancer cells [26]. Safer et al. prepared and characterized 414 nm Au/Ch/EGCG nanoconjugates that might be used in the hepatic fibrosis disorder treatment [27].

AuNPs were synthetized by the Turkevich method and then functionalized with cysteamine CystAuNPs [2]. Some studies suggested the positive therapeutic effects for cancer [8,19,28]. Fujisawa et al. reported cysteamine, an antioxidant aminothiol, to inhibit metastasis of pancreatic cancer [19]. Wan et al. showed the increase in doxorubicin chemotherapeutic effect by the autophagy-modulatory role of cysteamine in cancer cells [28]. Inano et al. suggested cysteamine as an inhibitor of estrogen receptor development of mammary tumors in rats [29]. Rubin et al. validated that cysteamine inhibits pancreatic cancer [20].

## 2. Materials and Methods

### 2.1. Materials

Epigallocatechin gallate (MW 458.372 g/mol) was obtained from Taiyo Kagaku. Chitosan (MW 250 kDa and degree of deacetylation > 93%) was acquired from Altakitin (Lisbon, Portugal). Trisodium hydroxide and tetrachloroauric (III) acid (HAuCl_4_; 99.99% trace metals basis, 30 wt% in dilute HCl), acetic acid, dimethyl sulfoxide (DMSO), N-Hydroysulfosuccinimide sodium salt (MW 217.13 g/mol), n-(3-Dimethylaminopropyl)-N′-ethyliarbodiimied hydrochloride (MW 191.70 g/mol), tween 80, sulforhodamine B (SRB), and trypan blue were purchased from Sigma-Aldrich (Darmstadt, Germany). Phosphate buffered saline (PBS: 0.01 M, pH 7.4) was purchased from Fluka (München, Germany). The antioxidant kit assay kit (Cayman Chemical Company) was obtained from Cayman Chemical CompanyMaster-InVitro (Barcelona, Spain). Regenerated cellulose dialysis membrane (8–10 kD) was acquired from Spectra/Por^®^. Amicon ultra (3 k, Merck KgaA) was obtained from Millipore S.A.S. (Molsheim Cedex, France). Cysteamine (MW 77.15 g/mol) was obtained from Selleck Chemicals LLC (Houston, TX, USA). BxPC-3 CRL-1687 cells was purchased from LGC standards, S.L.U. (Barcelona, Spain). Fetal bovine serum (FBS), phosphate-buffered saline (PBS), trypsin, Roswell Park Memorial Institute Medium (RPMI 1640 Medium) were obtained from Invitrogen Co. (Scotland, UK).

### 2.2. Conjugation of EGCG with Gold Nanoparticles Synthetized with Chitosan (EGCG-ChAuNPs)

An aqueous solution of acid chloroauric (1.0 mM, 1.0 mL) was mixed with a diluted solution of chitosan (5.0 mL, 0.32 *w*/*v*%) under magnetic stirring [30]. The mixture was heated for 30 min until a red suspension was obtained and ChAuNPs prepared. The concentration of the colloidal suspension ChAuNPs was 31.8 nM as calculated by the Lambert–Beer Law with a molar absorptivity of the AuNPs of 2.33 × 10^8^ M^−1^ cm^−1^ (plasmon resonance band at 526 nm) [19,31].

A total of 2.0 mL ChAuNPs were then conjugated with 1.0 mL of 0.27 mM EGCG via carbodiimide-mediated cross-linking (EDC/NHSS) with a molar ratio EGCG: EDC/NHSS of 1:0.5, at room temperature (RT) for one hour. The final solution was centrifuged at 12,000 rpm during 8 min to remove the excess of EGCG. The concentration of the EGCG-ChAuNPs was 14.5 nM (Lambert–Beer Law, Figure S1A).

### 2.3. Conjugation of EGCG with CystAuNPs (EGCG-CystAuNPs)

AuNPs were synthesized by the Turkevitch method. An aqueous solution of acid chloroauric (25 mL water, 17.2 μL HAuCl_4_) was mixed with a diluted solution of sodium citrate (2.5 mL, 0.03 g). The reaction time was 10 min under magnetic stirring and heating. Then, the AuNPs were functionalized with cysteamine with a molar ratio cysteamine: AuNPs of 1:1 [15,32]. The solution was stirred for 30 min at RT. The final nanoparticles concentration was 11.5 nM.

A total of 1.0 mL of 0.27 mM EGCG was conjugated with 5.0 mL of CystAuNPs via EDC/NHSS coupling reaction (with a molar ratio EGCG: EDC/NHSS of 1:01), under magnetic stirring, at room temperature. After, tween 80 (0.11 g) was added to the nanoparticles to enhance their stability, reacting during 15 min. The final solution was centrifuged at 12,000 rpm, for 8 min to remove the unbound EGCG. The estimated EGCG-CystAuNPs concentration was 1.36 nM (Figure S1B).

### 2.4. Dynamic Light Scattering

The hydrodynamic diameter (dynamic light scattering, DLS) and the zeta potential (laser Doppler velocimetry, LDV) of the nanoparticles in suspension were investigated using a Zetasizer Nano ZS (Malvern Instruments Ltd., Malvern, UK), at 25 °C. The size distribution was determined in a 12 mm square polystyrene cuvette (DTS 1060, Sarstedt, Nümbrecht, Germany), at a scattering angle of 173°.

### 2.5. Transmission Electron Microscopy (TEM)

TEM images were obtained using a Jeol JEM-1400, JEOL operated at 60 kV. A total of 5 μL of sample was placed on a carbon formvar-coated grid for 5 min and washed to remove the excess.

### 2.6. UV-Vis Spectroscopy

UV-Vis absorption spectra of the samples—AuNPs, ChAuNPs, CystAuNPs, EGCG-ChAuNPs and EGCG-CystAuNPs—were analyzed by Shimadzu UV-1700 PharmaSpec spectrophotometer, at RT.

### 2.7. EGCG Encapsulation Efficiency

The UV-Vis absorption spectra of the supernatant were determined, using Shimadzu UV-1700 PharmaSpec spectrophotometer, and the EGCG encapsulation efficiency (EE) of the nanoconjugates was calculated using Equation (1):

(1) $$\mathrm{EE}\left(\%\right)=\frac{\mathrm{Total}\;\mathrm{EGCG}\;\mathrm{in}\;\mathrm{suspension}-\mathrm{EGCG}\;\mathrm{in}\;\mathrm{supernatant}}{\mathrm{Total}\;\mathrm{EGCG}\;\mathrm{in}\;\mathrm{suspension}}\times 100$$

### 2.8. Attenuated Total Reflectance-Fourier Transform Infrared Spectroscopy (ATR-FTIR)

The suspensions of ChAuNPs, EGCG-ChAuNPs, CystAuNPs and EGCG-CystAuNPs were evaluated by ATR-FTIR spectroscopy with an ALPHA FTIR Spectrometer (Bruker) in the spectral range 4000–400 cm^−1^, resolution of 4 cm^−1^ and 64 scans, at RT.

### 2.9. In Vitro Release Studies

EGCG-ChAuNPs and EGCGCys-AuNPs release profiles were achieved in DI water. The samples were incubated with constant magnetic stirring, at 37 °C in regenerated cellulose dialysis membranes. Figure S2 displays the lowest and the highest in vitro typical release spectra corresponding to C/CEGCG.

### 2.10. Antioxidant Assay

The antioxidant capacity of the systems EGCG-ChAuNPs and EGCG-CysAuNPs was measured using the Cayman’s antioxidant assay kit.

### 2.11. Cell Culture

Human pancreatic cancer cell lines (BxPC3) were maintained in an RPMI medium, complemented with 10% FBS, at 37 °C under 5% CO_2_ humidified atmosphere.

### 2.12. In Vitro Cytotoxicity Evaluation

The effects of the nanosystems and EGCG alone on the BxPC3 cell growth were assessed by the Sulforhodamine B (SRB) method.

A total of 1000 BxPC3 cells per well were seeded under 5% CO_2_ humidified atmosphere, at 37 °C. Cells were treated with the control samples and samples. The controls are CystAuNPs and ChAuNPs at the concentrations ranging between 0 and 1.0 nM for 24 h. The samples are EGCG, EGCG-CystAuNPs and EGCG-ChAuNPs with EGCG concentrations between 2.0 and 25 μM. Following the 24 h incubation, the cells fixated with 10% TCA for 1 h on ice, were washed and stained with 50 µL SRB dye for 30 min. After, 1% acetic acid was used to the wash the cells. The samples were dried, and the protein-bound stain was solubilized with 10 mM Tris solution. The SRB absorbance was evaluated at 560 nm using the PowerWave microplate reader. Figure S5 exhibits the lowest and the highest cell growth results of the BxPC3 after 24 h incubation with the nanoparticles alone. Figure S6 shows the lowest and the highest growth results after incubation with free EGCG, EGCG-AuChNPs and EGCG-CysAuNPs. The IC50 concentrations of EGCG, EGCG-CystAuNPs and EGCG-ChAuNPs were estimated.

### 2.13. Caspase-3 Activation Assay

The nanosystems were dissolved in PBS buffer and incubated with BxPC3 cells at an EGCG concentration of 5 μM for 24 h. The toxicity of the samples was analyzed by the activation of caspase-3. Briefly, confluent BxPC3 were incubated with EGCG, EGCG-ChAuNPs and EGCG-CystAuNPs for 24 h. The cells were tripsinized and centrifuged. The pellet of cells was lysed in 100 μL hypotonic lysis buffer. To estimate caspase-3 activation, 40 μL of the lysate solution was used to achieve the total cellular protein concentration at 595 nm. Figure S7 exhibits the lowest and the highest results obtained for caspase-3 activation assay.

### 2.14. Statistical Analysis

Three independent experiments were measured. Statistical significance (*p* < 0.05) was determined by the Student’s *t*-test.

## 3. Results and Discussion

**Figure 1:**
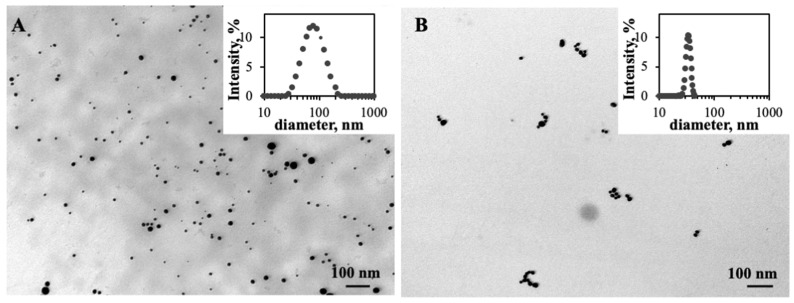
TEM image of: (**A**) ChAuNPs and (**B**) CystAuNPs. The scale bar is 100 nm.

TEM images (Figure 1) present the ChAuNPs and CystAuNPs formulated. A polydisperse ChAuNPs suspension was synthesized through the reduction in gold salt by chitosan presenting a spherical shape (Figure 1A). These results corroborate intensity distribution graph and size analyzed by DLS measurements (Table 1). CystAuNPs were prepared by the functionalization with cysteamine of gold nanoparticles synthesized by the Turkevitch method [33]. CystAuNPs (Figure 1B) have mainly spherical shapes.

The hydrodynamic diameters and the zeta potential values of the nanoparticles are summarized in Table 1. A total of 86 ± 16 nm ChAuNPs were obtained with a zeta potential of approximately 22 mV, suggesting good stability of NPs in the colloidal suspension, which corroborates the literature [33,34]. Chitosan acts as a reducing agent of gold as well as a stabilizer. The molecular weight and the degree of deacetylation (DD) of the chitosan present an important role in the nanoparticles hydrodynamic diameter [35]. In fact, Abrica-González et al. prepared 5 nm of chitosan AuNPs with a zeta potential of 43.3 mV, which is significantly different from the produced nanoparticles of this study. Chitosan with low Mw and DD allows the fabrication of stable chitosan nanoparticles with small diameters. The average of the functionalized CystAuNP hydrodynamic diameters is 54 ± 9 nm, a significant modification on the AuNPs size (37 nm) that could indicate the efficient functionalization of the nanoparticles. Their zeta potential is −37 mV, suggesting good stability of the colloidal suspension.

**Figure 2:**
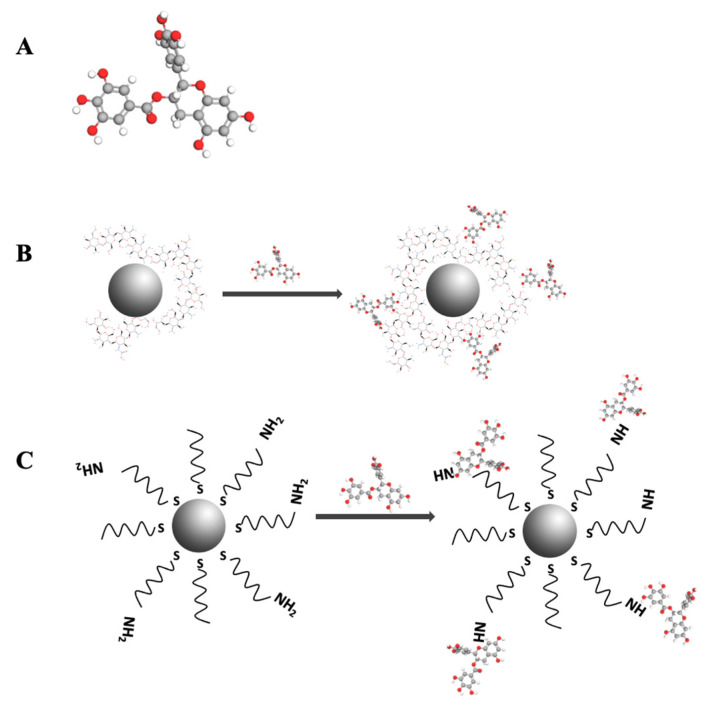
(**A**) Chemical structure of EGCG; (**B**) Scheme of nanoconjugate EGCG-ChAuNPs; (**C**) Scheme of nanoconjugate EGCG-CystAuNPs.

The conjugation of ChAuNPs with EGCG was developed via carbodiimide-mediated cross-linking of carboxylic acids (Figure 2B). The hydrodynamic diameter is 125 ± 13 nm and the average zeta potential is 36 ± 6 mV in water. ChAuNPs tend to form some stable aggregates due to the electrostatic attractive forces between amino groups in Ch and AuCl_4^−^_ in suspension (zeta potential is 22 ± 5 mV) [36,37].

**Figure 3:**
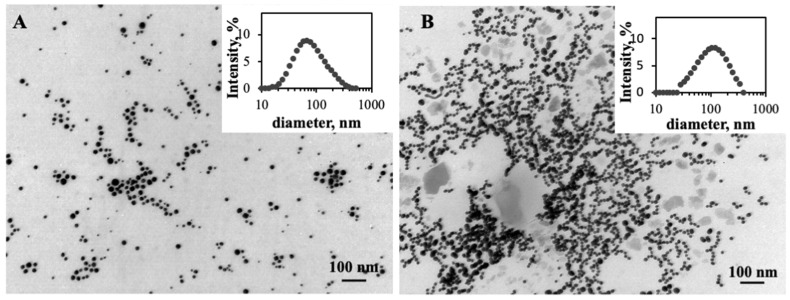
TEM image of: (**A**) EGCG-ChAuNPs; (**B**) EGCG-CystAuNPs. Scale bar is 100 nm.

The polydispersity of the ChAuNPs occurs due to the degree of deacetylation of chitosan that promotes the increase in the PdI [24]. TEM analysis (Figure 3A) confirmed the spherical shape of the nanoparticles.

The synthesized EGCG-CystAuNPs (Figure 3B) present 111 ± 1 nm of diameter and a zeta potential of −24 ± 1 mV. The high AuNPs reactivity surface due to the EDC/NHSS coupling reaction might be the reason for the size increase.

**Figure 4:**
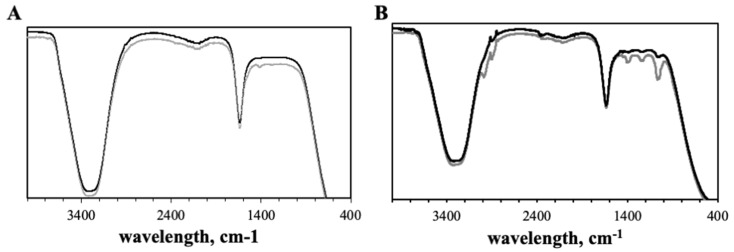
(**A**) FTIR spectra of ChAuNPs (grey line) and EGCG-ChAuNPs (black line). (**B**) FTIR spectra of CystAuNPs (grey line) and EGCG-CystAuNPs (black line). The spectra were shifted for a better visualization.

Figure 4 displays the nanoparticles ATR-FTIR spectra. To confirm the chemical composition of the prepared nanosystems, ATR-FTIR spectra of EGCG, ChAuNPs, CystAuNPs, EGCG-ChAuNPs and EGCG-CystAuNPs were obtained.

ChAuNPs spectrum demonstrates absorption bands at 3335 cm^−1^ (amine N–H symmetric vibration and O–H group), at 2920 cm^−1^ from the C–H group and at 1668 cm^−1^ and 1553 cm^−1^ from the C–O stretching along with the N–H deformation mode (acetylated amine and to free amine groups) (Figure 4A). The N–H bending suggests that the chitosan acts as stabilizer and reducing agent on the synthesis of AuNPs as reported by Abrica-González et al. [33]. The CH_3_ vibrations are presented at 1421 cm^−1^ and at 1296 cm^−1^; the C–N bond is observed at 1301 cm^−1^. The spectra of conjugated EGCG-ChAuNPs (Figure 4A and Figure S4A, black line) shows a peak at 3290 cm^−1^ assigned to the amine group. At 1553 cm^−1^ the C–O stretching along with the N–H deformation mode disappeared. A chemical modification by EDC/NHSS is suggested and it formed an amide bond covalently from ChAuNPs and polyphenol EGCG (at 1661 cm^−1^, 1534 cm^−1^ and 1460 cm^−1^ visualized on Figure S3) [34]. On Figure 4B and Figure S4B, the CystAuNPs ATR-FTIR spectrum exhibited characteristic frequencies of 3653 cm^−1^ and 3671 cm^−1^ from the N–H stretch bond of primary amine. An intensive absorption band at 2988 cm^−1^ indicates C–H of alkanes. The 2903 cm^−1^ wavenumber corresponds to a C–H stretch of aldehyde. The peak at 1661 cm^−1^ and 2051 cm^−1^ might indicate the salt formed by CSH with HCl (−N$H_{3}^{+}$). The 1409 cm^−1^ band corresponds to the strong symmetric vibration of the νC=O (1245 cm^−1^), which indicates the stretching vibration of C–N bond. In the spectrum of EGCG-CystAuNPs, the wavenumber 1542 cm^−1^ indicates the N–H bond from amide linked by alcohol group of polyphenol EGCG and primary amine of the nanoparticles, via EDC/NHSS coupling reaction (Figure S4B) [23]. The C–H group of aromatic ring at 845 cm^−1^ was observed.

EGCG conjugation efficiency was determined for both nanosystems, by UV-Vis spectroscopy. A total of 60 ± 2% and 78 ± 1.3% values of EGCG encapsulation efficiency achieved to EGCG-ChAuNPs and EGCG-CystAuNPs, respectively.

The antioxidant activity of EGCG conjugated with ChAuNPs and CystAuNPs was studied and presented in Table 2. The free radical scavenging activity of the produced nanosystems was assessed by the ABTS method.

According to the results, EGCG-ChAuNPs and EGCG-CystAuNPs present high capacity to scavenge the ABTS generated in aqueous phase. This suggested that EGCG could be protected by the nanoparticles and the antioxidant activity of EGCG was preserved. Additionally, the antioxidant capacity of the EGCG might be improved by the conjugation with AuNPs.

The stability of EGCGChAuNPs and EGCGCystAuNPs was evaluated in terms of hydrodynamic diameter and zeta potential, in pH 5.3 and pH 7.4 at 37 °C, in the dark for 60 days. Data suggested stable EGCGChAuNPs for 24 h and for 7 days at pH 7.4 and pH 5.3, respectively. EGCGCystAuNPs presents higher stability at pH 7.4 and 37 °C (60 days) than at pH 5.3 and 37 °C (2 days), suggesting that the high electrostatic repulsion is responsible for the maintenance of the size of the nanoparticles.

**Figure 5:**
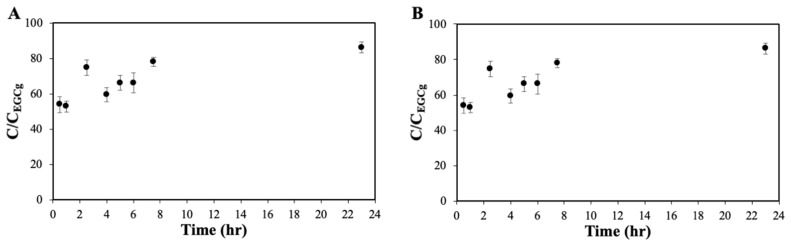
In vitro release profiles of (**A**) EGCG-ChAuNPs and (**B**) EGCG-CystAuNPs at pH 7.4 (in PBS 0.01 M) at 37 °C. C_EGCG_ corresponds to the total amount of EGCG added.

The in vitro release kinetics from nanoconjugates were performed, using dialysis membranes in PBS solution at 37 °C and pH 7.4. The conjugated EGCG exhibits a fast release from ChAuNPs (Figure 5A). This fact might occur due to part of EGCG being absorbed in the nanoparticles surface.

In fact, after 30 min, 54% of EGCG was released from EGC-ChAuNPs nanoconjugates (Figure 5A). In 8 h, 52% of the EGCG was released from EGCG-CystAuNPs (Figure 5B). Comparing both nanoconjugates, after 24 h of incubation, 86.2% and 60.9% of the EGCG was released from ChAuNPs and CystAuNPs, respectively. These data correspond to 0.038 and 0.041 mM of EGCG concentration in CystAuNPs and ChAuNPs suspensions, respectively. The complete amount of catechin was achieved after 48 h.

**Figure 6:**
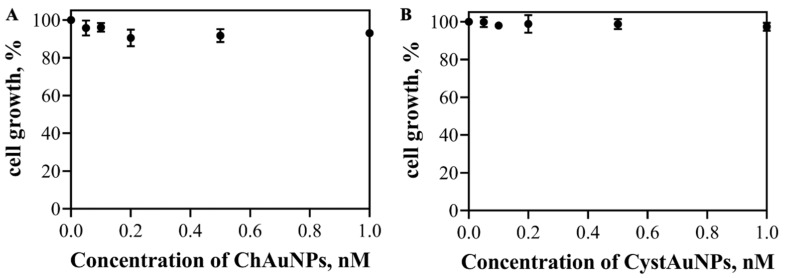
Effect of ChAuNPs (**A**) and CystAuNPs (**B**) on the cell growth of pancreatic cancer BxPC3 cells.

The cytotoxicity of ChAuNPs and CystAuNPs to BxPC3 cells was evaluated by the SRB method. Figure 6 displays the cell growth results of the cancer cells after 24 h of incubation with the nanoparticles alone. Figure 5 shows that, for concentrations up to 1 nM, CystAuNPs do not have effect on the cell growth of BxPC3 cells. For ChAuNPs, concentrations up to 2 nM the same effect was verified. For concentrations above 0.2 nM until 1 nM, ChAuNPs present 10% of toxicity on this type of cancer cells.

**Figure 7:**
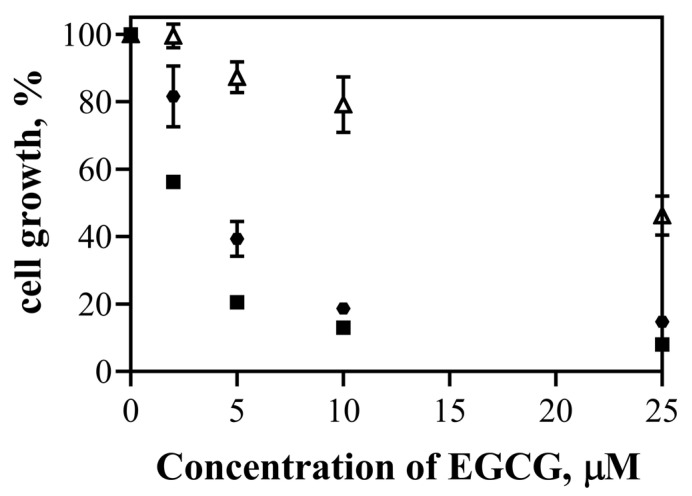
Effect of free EGCG (Δ), EGCG-AuChNPs (■) and EGCG-CysAuNPs (⬣) on the growth of pancreatic cancer (BxPC3 cells).

The cytotoxic effect of EGCG and the nanosystems (EGCG-ChAuNPs and CystAuNPs) was studied against the same cancer cell line, the BxPC3 cells (Figure 7).

The cell growth of BxPC3 incubated with EGCG alone was found to be in the range of 99.6–46.3% for the EGCG concentration of 2.0–25.0 μM. For the same concentrations, it presented cell growth in the range of 56.2–8.0% and 81.6–14.7% when incubated with EGCG-ChAuNPs and EGCG-CystAuNPs, respectively. These values demonstrated a significant effectiveness of both nanoconjugates comparing with EGCG alone, as visualized by IC_50_ in Table 3. In particular, for an EGCG concentration of 5 and 10 μM, the cytotoxicity of conjugated EGCG (both nanosystems) was significantly higher than EGCG alone. The cell growth of BxPC3 decreases from 87.3% incubated with 5 μM EGCG alone, to 20.5% and 39.3% when incubated with EGCG-ChAuNPs and EGCG-CystAuNPs, respectively. These results are in accordance with the achieved antioxidant activity of the nanoconjugates as well as the in vitro release profiles of the EGCG-ChAuNPs and EGCG-CystAuNPs. In fact, the nanoconjugates presented a high antioxidant capacity that could be reliable for the decrease in the BxPC3 cell growth. Additionally, the data corroborated the literature and the enhancement of the chemical activity of EGCG when conjugated on the gold nanosurface might occur [17].

**Figure 8:**
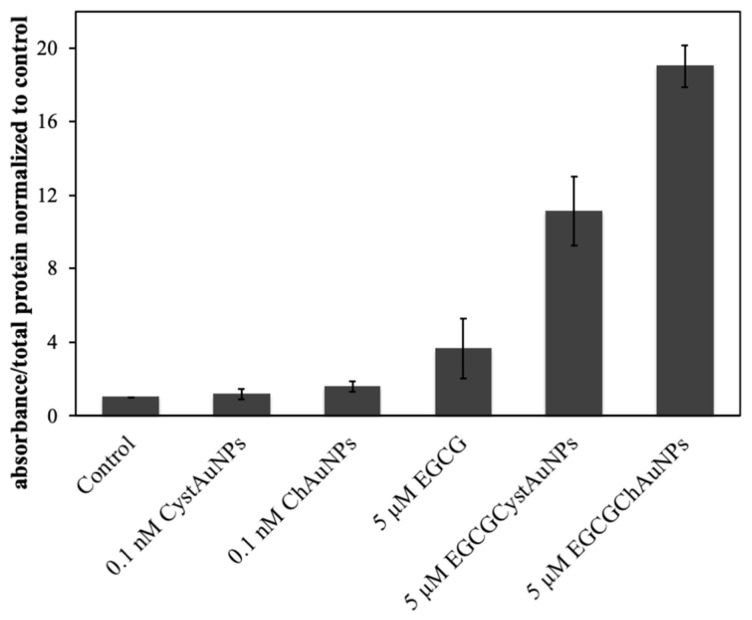
Activation of caspase-3 in BxPC3 cells exposed for 24 h to free EGCG, EGCG-CystAuNPs, EGCG-ChAuNPs, CystAuNPs and ChAuNPs.

Conjugated EGCG significantly induced apoptosis in BxPC3 as evidenced by the increase in caspase-3 activity when compared with non-treated BxPC3 cells and cells treated with ChAuNPs and CystAuNPs at 0.1 nM of concentration (Figure 8). For EGCG-ChAuNPs, this evidence is more pronounced.

## 4. Conclusions

This work proposes two methodologies to prepare nanocarriers based on gold nanoparticles decorated with EGCG. We demonstrated that the EGCG conjugates nanosystems enhance the inhibition of BxPC3 cell growth than EGCG alone. It is possible to decrease the EGCG-ChAuNPs and EGCG-CystAuNPs concentration 10.5 and 6.4 times to have the same effect as the EGCG alone. This effect is more pronounced by the positive charged EGCG-ChAuNPs. This fact might promote the presence of attractive forces, resulting in an enhanced accumulation and BxPC3 cell uptake.

In summary, these results suggest that the effectiveness of EGCG conjugated NPs for cancer therapy increase the antioxidant efficacy and, therefore, the cell growth inhibition. This study could provide promising delivery systems with an encapsulation efficiency above 60% and the possibility of scaling up the process for the pancreatic cancer treatment. The nanoparticles were nontoxic to BxPC3 cancer cells proposing that the AuNPs could be produced as a chemopreventive vector. In vitro assays showed that EGCG-ChAuNP and EGCG-CystAuNP nanoconjugates present 50% inhibitory concentration 10.4 and 6.2 times lower than the EGCG alone, respectively. To have a better understanding of the concept based on these systems, other type of cancer cells and even normal cell lines should be tested.

## Figures and Tables

**Table 1:** Hydrodynamic diameter, polydispersity index (PdI) and zeta potential of AuNPs, CystAuNPs, EGCG-CystAuNPs, ChAuNPs, EGCG-ChAuNPs.

Samples	Size, nm	PdI	Zeta Potential, mV
AuNPs	37 ± 1	0.2	−33 ± 2
CystAuNPs	54 ± 9	0.6	−37 ± 3
EGCG-CystAuNPs	111 ± 1	0.2	−24 ± 1
ChAuNPs	86 ± 16	0.2	22 ± 5
EGCG-ChAuNPs	125 ± 13	0.3	36 ± 6

AuNPs: gold nanoparticles; ChAuNPs: chitosan-gold nanoparticles; EGCGChAuNPs: conjugated chitosan-gold nanoparticles with EGCG; CystAuNPs: gold nanoparticles functionalized with cysteamine; EGCGCystAuNPs: Conjugated CystAuNPs with EGCG (*n* = 3).

**Table 2:** ABTS radical scavenging activity of ChNPs, CystAuNPs, EGCG-ChAuNPs and EGCG-CystAuNPs.

	Antioxidant Activity (mM)			
Wavelength (nm)	AuChNPs	CystAuNPs	EGCG-ChAuNPs	EGCG-CystAuNPs
750	-	1.8	2.5	3.5

**Table 3:** Half maximal inhibitory concentration (IC_50_) values on BxPC3 cells.

	Free EGCG	EGCG-ChAuNPs	EGCG-CystAuNPs
IC_50, μM_	23 ± 3	2.22 ± 0.02	3.7 ± 0.2

## Supplementary Materials

The following are available online at https://www.mdpi.com/article/10.3390/pharmaceutics14030491/s1, Figure S1: UV-vis absorption spectra of: (A) EGCG-ChAuNPs and (B) EGCG-CystAuNPs, Figure S2: In vitro release spectra of (A) EGCG-ChAuNPs and (B) EGCG-CystAuNPs at pH 7.4 (in PBS 0.01 M) at 37 °C. • corresponds to the lowest release data; • corresponds to the highest release data. CEGCG corresponds to the total amount of EGCG added, Figure S3: FTIR spectra of EGCG. The spectra were shifted for a better visualization, Figure S4: FTIR spectra of: (A) EGCG-ChAuNPs and (B) EGCG-CystAuNPs. The spectra were shifted for a better visualization. The final spectra correspond to the subtraction of deionized water FTIR spectrum to the nanoconjugates FTIR spectra, Figure S5: Effect of ChAuNPs (A) and CystAuNPs (B) on the cell growth of BxPC3 cells. -▲- and -▲- correspond to the lowest and highest cell growth values, respectively, Figure S6: Effect of free EGCG (▲ and ▲), EGCG-AuChNPs (■ and ■) and EGCG-CysAuNPs (• and •) on the growth of pancreatic cancer. The grey and black symbol correspond to the lowest and highest cell growth data, respectively, Figure S7: Activation of caspase-3 in pancreatic cancer cells exposed for 24 h to free EGCG, EGCG-CystAuNPs, EGCG-ChAuNPs, CystAuNPs and ChAuNPs. Grey and black column correspond to the lowest and highest absorbance/total protein normalized to control, respectively.
