# Supplementary material for: Nanocarriers Based on Gold Nanoparticles for Epigallocatechin Gallate Delivery in Cancer Cells
Source: Pharmaceutics. 2022 Feb 24;14(3):491. doi: 10.3390/pharmaceutics14030491 (PMC8951757; doi:10.3390/pharmaceutics14030491)
Supplement: Supplementary file 1 [file pharmaceutics-14-00491-s001.zip › pharmaceutics-1561009-supplementary.pdf]

pharmaceutics

Supplementary

Nanocarriers Based on Gold Nanoparticles for Epigallocatechin Gallate Delivery in Cancer Cells

Lídia Cunha, Sílvia Castro Coelho, Maria do Carmo Pereira and Manuel A. N. Coelho

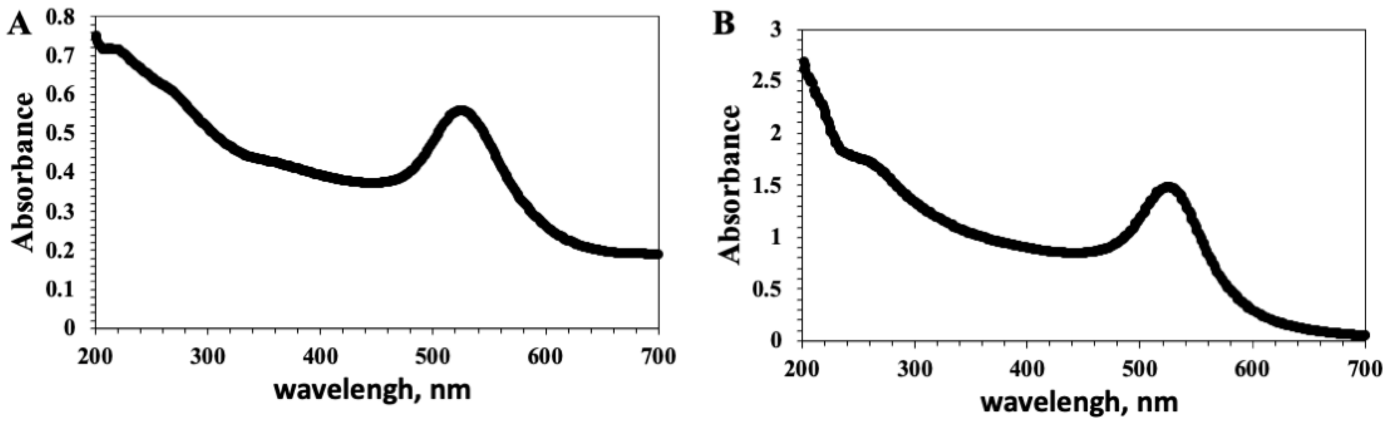

Figure S1. UV-vis absorption spectra of: A) EGCG-ChAuNPs and B) EGCG-CystAuNPs.

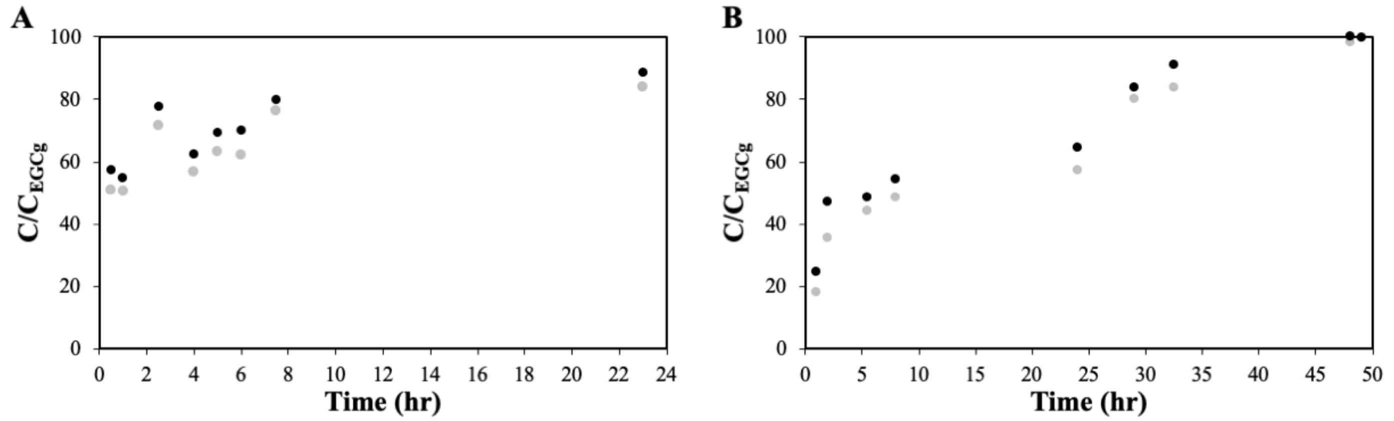

Figure S2. *In vitro* release spectra of A) EGCG-ChAuNPs and B) EGCG-CystAuNPs at pH 7.4 (in PBS 0.01M) at 37ºC. • corresponds to the lowest release data; • corresponds to the highest release data. C_{EGCG} corresponds to the total amount of EGCG added.

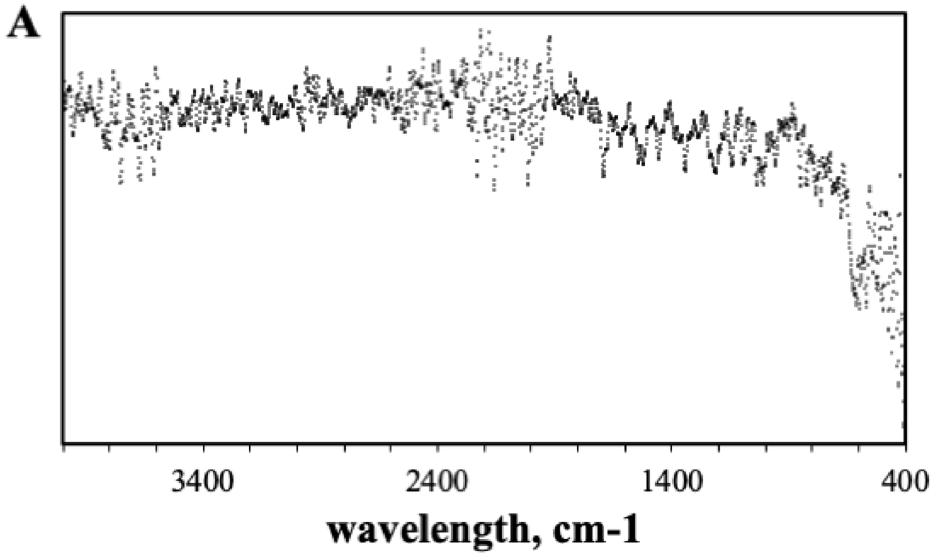

Figure S3. FTIR spectra of EGCG. The spectra were shifted for a better visualization.

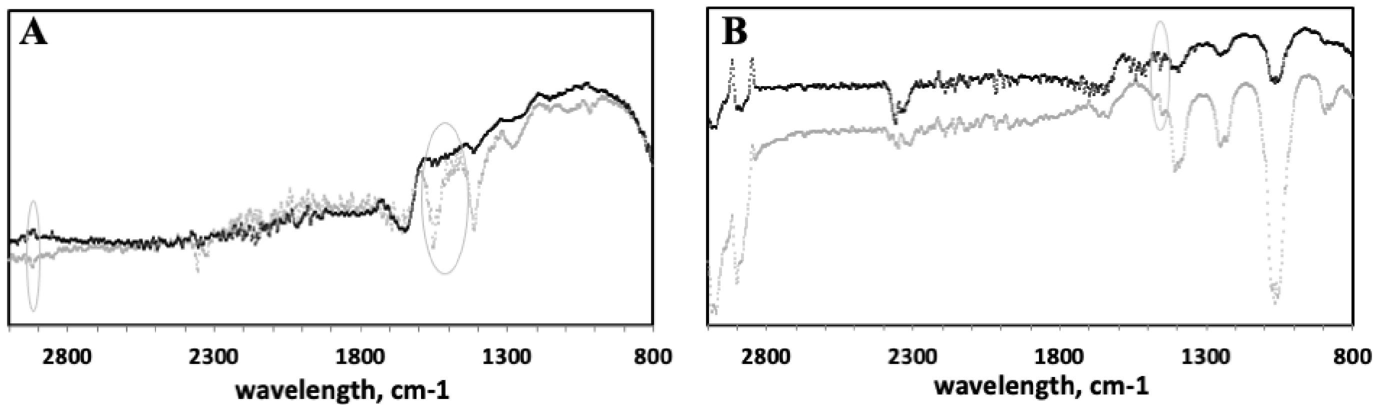

Figure S4. FTIR spectra of: A) EGCG-ChAuNPs and B) EGCG-CystAuNPs. The spectra were shifted for a better visualization. The final spectra correspond to the subtraction of deionized water FTIR spectrum to the nanoconjugates FTIR spectra.

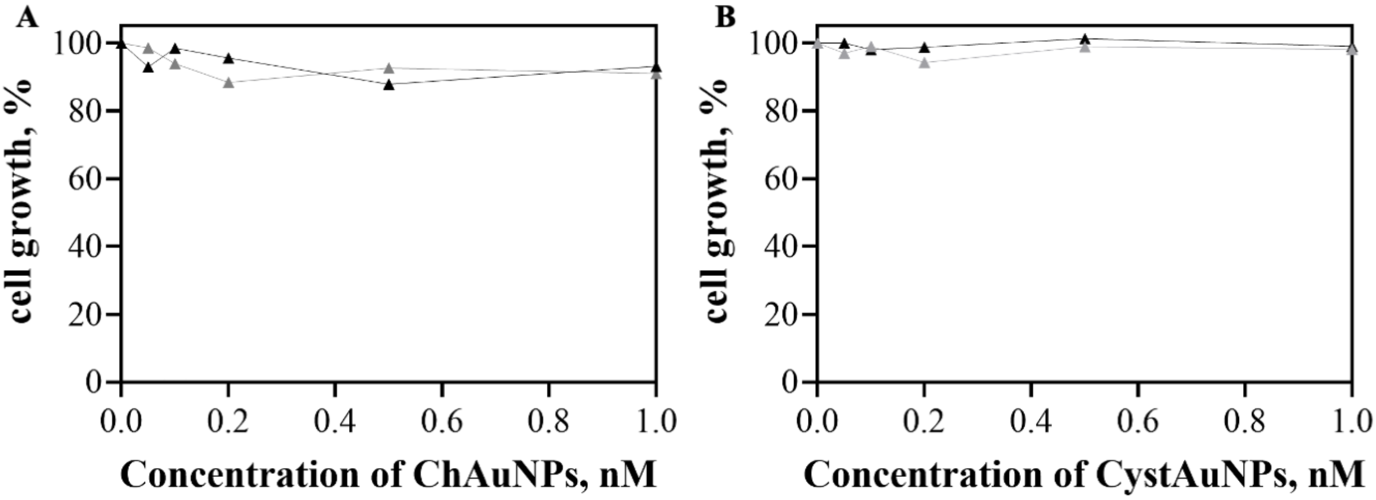

Figure S5. Effect of ChAuNPs (A) and CystAuNPs (B) on the cell growth of BxPC3 cells. -▲- and -▲- correspond to the lowest and highest cell growth values, respectively.

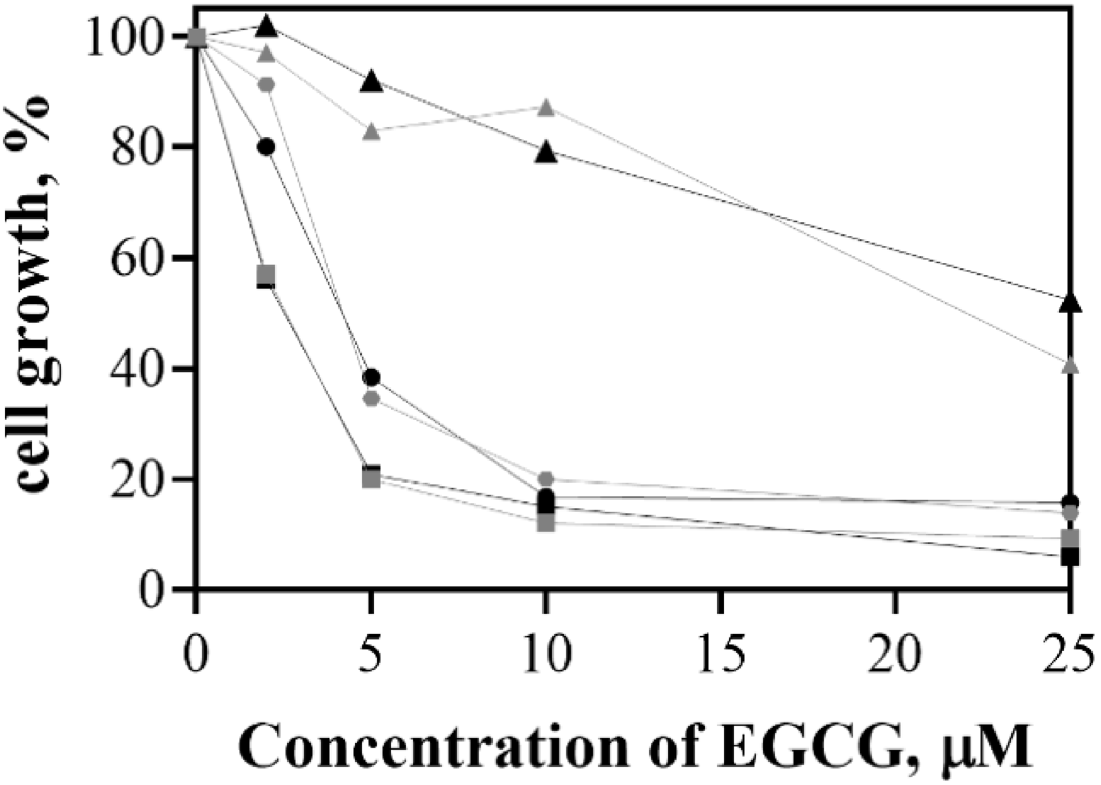

Figure S6. Effect of free EGCG (▲ and ▲), EGCG-AuChNPs (■ and ■) and EGCG-CysAuNPs (• and •) on the growth of pancreatic cancer. The grey and black symbol correspond to the lowest and highest cell growth data, respectively.

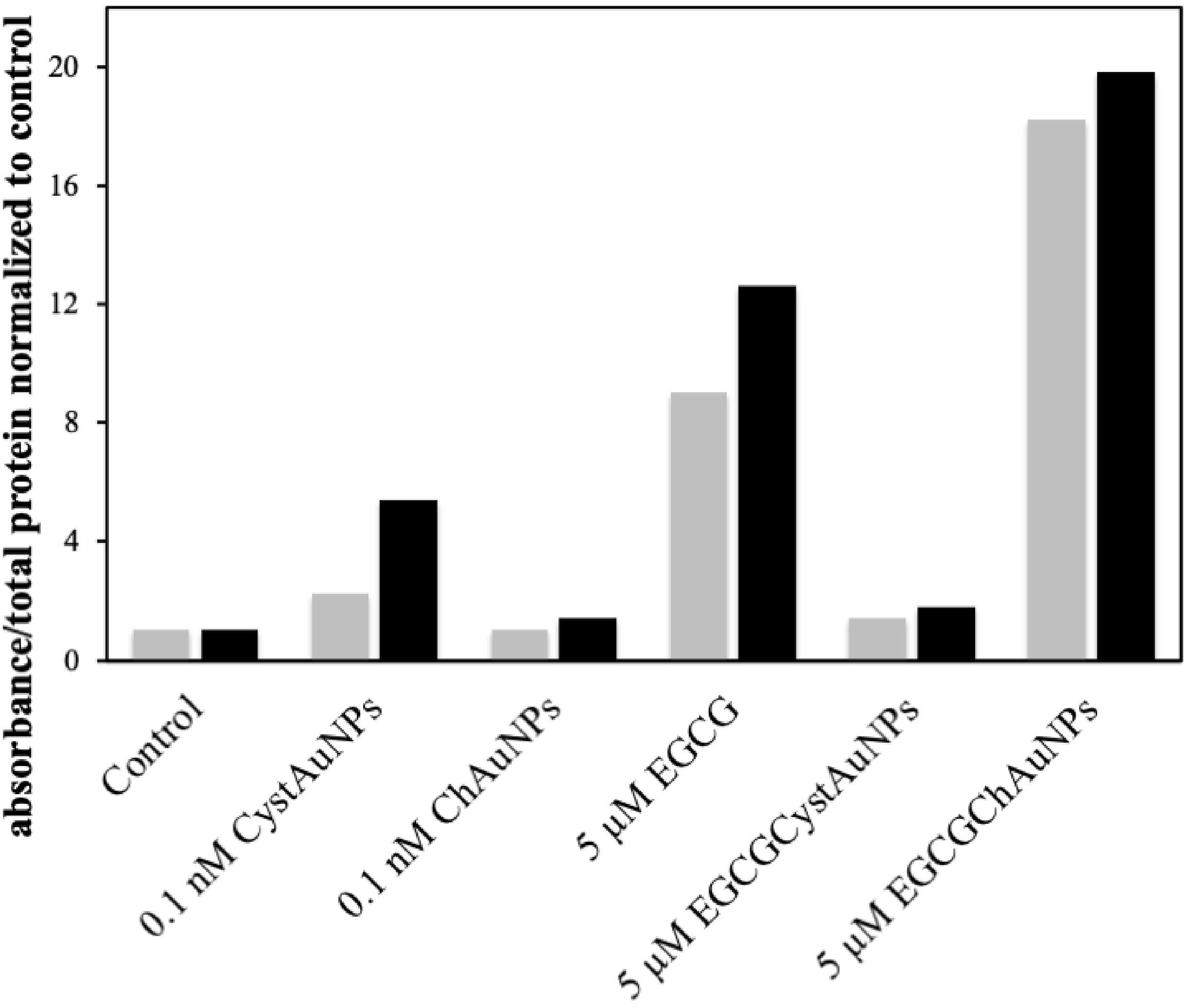

Figure S7. Activation of caspase-3 in pancreatic cancer cells exposed for 24 hours to free EGCG, EGCG-CystAuNPs, EGCG-ChAuNPs, CystAuNPs and ChAuNPs. Grey and black column correspond to the lowest and highest absorbance/total protein normalized to control, respectively.
